# Why malnutrition in orthopaedic elective patient is still an issue? A recent review of the literature

**DOI:** 10.1007/s00590-023-03593-z

**Published:** 2023-05-31

**Authors:** Marco Pes, Alessio Pulino, Francesco Pisanu, Andrea Fabio Manunta

**Affiliations:** Orthopaedic Department, University of Sassari, Viale San Pietro 43, 07100 Sassari, Italy

**Keywords:** Elective surgery, Arthroplasty, Malnutrition, Risk factor

## Abstract

**Background:**

Malnutrition is a known risk factor for complications and adverse outcomes after elective total joint arthroplasty (TJA). The progressive increase in the ageing of world population and in the numbers of TJA, widens the demand for a faster post-operative recovery and function. The aim of this study was to review the literature regarding: post-operative transfusion, infections, wound complications, length of hospital stay (LOS), rate of admission in intensive care unit (ICU), and total patient charges, in malnourished patient undergoing TJA.

**Methods:**

The search reviewed all fields of the available peer-reviewed literature, published in the English language during the last seven years 2015–2022. We started from a total of 745 studies and finally we included in the review 16 articles.

**Results:**

In 10 studies, an increased surgical site infection was shown, being by far the most common complication, in 8 studies, malnutrition was associate with the increase of the average length of stay (LOS), and in 5 studies, the major founding was the increase in costs. An increase of the morbidity was found in 3 studies, instead a larger number of transfusions was highlighted in 2 studies. Lastly, one study showed a major unplanned ICU admission rate.

**Conclusions:**

Although the literature trend indicates that the nutritional status of TJA candidate patients is a parameter that influences the surgical outcome, in particular surgical site infections, length of stay, and costs, there are, to the authors’ knowledge, no studies aimed at identifying validated and recognized protocols for the correction of malnutrition.

## Introduction

Malnutrition is a known risk factor for complications and adverse outcomes after elective total joint arthroplasty (TJA), but little is known about the burden this risk factor places on the health care system [1]. It is widespread regarding hospitalized patients in the field of orthopaedic surgery and results in suboptimal clinical outcome [2]. A growing body of evidence correlate malnutrition with several adverse outcomes; however, to date a few study have demonstrated an improvement in post-operative outcomes following TJA with a focus on nutritional intervention for patients with pre-operative hypalbuminaemia. For example, Schroer et al., in a prospective study, showed that joint arthroplasty outcomes were positively affected in study patients with low albumin when a high-protein, anti-inflammatory diet was encouraged, whereas elective surgery was neither cancelled nor delayed with a malnutrition designation [3].

From a metabolic perspective, surgery create a state of insulin resistance, inducing metabolic changes in glucose metabolism. Post-operative insulin resistance contributes to hyperglycaemia, therefore net protein catabolism, which is associated with clinical complications. The orthopaedic surgical insult results in many responses that cause a change in metabolism towards catabolism. There are many ways in which the catabolic responses can be reduced and anabolism supported. We strongly believe that nutritional care should be seen as an integral part of the optimal management of surgical patients. Pre-operative preparation, good surgery and anaesthesia, optimal post-operative analgesia and fluid balance, and early mobilization and feeding, form an integrated programme to “enhanced recovery after surgery” (ERAS) [4].

Furthermore, the progressive increase in the ageing of world population and in the numbers of TJA [5] widens the demand for a faster post-operative recovery and function, nutrition should be considered for its role in managing the surgical stress response. The aim of this study was to review the literature regarding: post-operative transfusion, infections, wound complications, length of hospital stay (LOS), rate of admission in intensive care unit (ICU), and total patient charges, in malnourished patient undergoing TJA [3, 6].

## Materials and methods

A review was conducted to evaluate the role of malnutrition in TJA procedures. This study was developed according to the preferred reporting items for systematic reviews and meta-analyses (PRISMA) statement (www.prismastatement.org/PRISMAStatement) [7]. A computer-based literature search was completed in May 2022, and the electronic databases reviewed included PubMed and Google Scholar. The search reviewed all fields of the available peer-reviewed literature published in the English language (or those where a translation was available) during the last seven years 2015–2022. The search strategy combined terms using Boolean operators related to the type of surgery (“knee arthroplasty”, “hip arthroplasty”, or “joint replacement”) with those related to exposure (“malnutrition”, “hypoalbuminemia”, or “albumin”) and outcomes (“infection” or “complication”). We found a total of 745 studies. Our inclusion criteria were the population, total hip replacement patients, and total knee replacement patients; nutritional assessment evaluated with albumin < 3.5 mg/Dl or NRS < 3; complications in malnourished patient examined surgical site infection, length of stay, ICU admission, transfusions, increase in costs, and morbidity; prospective studies and retrospective studies; published in English after 2015.

**Fig. 1:**
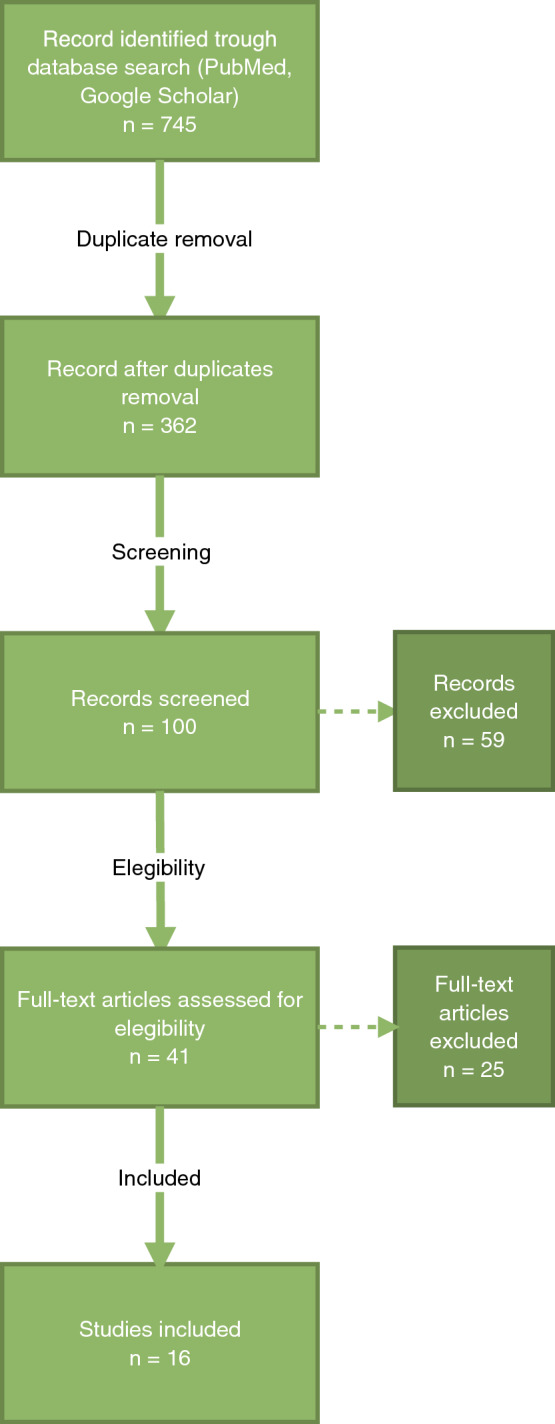
Flowchart

We started from a total of 745 studies, once removed 40 duplicates, a total of 705 studies were examined by two authors independently. The titles and abstracts of the studies were read and identified in the search, and excluded those that were irrelevant and that did not reflect the inclusion criteria. Therefore, the full text of the selected articles was scrupulously evaluated to determine whether it contained information on the topic of interest. Finally, we included in the review 16 articles (Fig. 1). In 14 of them, albumin value < 3.5 g/dl has been used as cut-off value to indicate malnourishment, and two studies use the NRS score < 3 (nutritional risk score) as nutritional assessment.

Quality assessment was not a factor for inclusion or exclusion within the review but was utilized to facilitate interpretation of findings.

## Results

In 10 studies, an increased surgical site infection was shown, being by far the most common complication, in 8 studies, malnutrition was associate with the increase of the average length of stay (LOS), and in 5 studies, the major founding was the increase in costs.

An increase of the morbidity was found in 3 studies, instead a larger number of transfusions was highlighted in 2 studies.

Lastly, one study showed a major unplanned ICU admission rate.

All the results are summarized in the table (Table 1) [1–3, 6, 8–19].

**Table 1:** All the authors used albumin level < 3.5 g/dl to identify malnourished patients, only one author used NRS ≥ 3

References	Complication	*p* value
Walls et al. [13]	+ Surgical site infection	< 0.001
	+ Morbidity	< 0.001
Kamath et al [18]	+ Unplanned ICU admissions	0.001
Bohl et al. [12]	+ Length of stay	< 0.001
	+ Surgical site infection	< 0.001
Kamath et al. [8]	+ Surgical site infection	< 0.001
	+ Transfusions	< 0.001
Ihle et al. [2]	+ Length of stay	
	+ Surgical site infection	
	+ Increase in costs	
Fu et al. [14]	+ Surgical site infection	< 0.001
	+ Morbidity	< 0.001
Rudasill et al. [10]	+ Increase in costs	0.001
Roche et al. [6]	+ Surgical site infection	
Ihle et al. [9]	+ Length of stay	0.001
	+ Surgical site infection	
Schroer et al. [3]	+ Length of stay	0.007
	+ Increase in costs	< 0.001
Fryhofer et al. [19]	+ Length of stay	< 0.001
	+ Morbidity	< 0.001
Black et al. [15]	+ Length of stay	< 0.001
	+ Surgical site infection	< 0.001
Bala et al. [1]	+ Increase in costs	< 0.001
Kishawi et al. [16]	+ Surgical site infection	< 0.005
Wilson et al. [11]	+ Length of stay	< 0.001
	+ Transfusions	< 0.001
	+ Surgical site infection	< 0.001
	+ Increase in costs	< 0.001
Eminovic et al. [17]	+ Surgical site infection	< 0.001

All the authors used albumin level < 3.5 g/dL to identify malnourished patients, besides Ihle, Christoph et al., which use NRS ≥ 3

## Discussion

The ageing of world population and the expected increase of TJA implants turned, once again, the spotlight on the nutritional status and the related complications, indeed the number of articles about malnutrition and orthopaedic patient increased in the last 6 years.

Malnutrition is defined as a state resulting from lack of uptake or intake of nutrition leading to altered body composition and diminished function. It is reported as 9–39% in hospitalized orthopaedic patient [20], and at an urban academic centre was identified in 26% of arthroplasty patients, but other studies report this incidence as high as 50% [21]. Little has been done to correct and prevent this important risk factor since 1987, when Smith TK et al. discussed the importance of the nutritional status of the orthopaedic patients undergoing surgery [20].

This review of the recent literature evaluated, which parameters, among post-operative transfusion, surgical site infection, length of hospital stay (LOS), rate of admission in intensive care unit (ICU), and total patient charges, are most affected in a malnourished patient undergoing TJA.

The definition of malnutrition had been difficult, due to the lack of a cohesive definition, a very important point of view on the argument has been outlined by Loftus et al., who specified that albumin serum level is only valid as marker of nutrition status, while at homeostasis, such orthopaedic elective patients undergoing TJA [8, 22]. Therefore, we decided to choose the studies, which took serum albumin < 3.5 g/dl, and NRS score < 3 (which has been proved to be comparable), as a marker of pre-operative malnutrition [2, 9].

The most widely used definition of surgical site infection has been provided by the Centers for Disease Control and Prevention (CDC) who classified it by depth and tissue spaces involved [23]. Prior studies have linked malnutrition to delayed wound healing and surgical site infection, and surely it is the parameters most widely analysed in the literature; in fact, even in our review, we found in the last 6 years, 10 studies, which strongly correlated surgical site infections with malnutrition, through several mechanism. All the studies analysed had a *p* value < 0.0001, suggesting to the authors’ opinion, that it is no longer justifiable to not study preoperatively the nutritional status of patient undergoing TJA.

However, TJA is a commonly, safe and well tolerate surgery for most patient; in same case, it can carry major and minor complications leading to an unplanned ICU admission [24]. One study statistically correlated malnutrition with a greater incidence of ICU compared to benchmark patients. Therefore, patients need to be counselled on this increased possibility, and physicians need to be aware of the forensic aspects involved, in fact Kamath et al. strongly suggested to postpone elective surgery until medical status is optimized [10]. An increased risk of transfusion is another parameter that was found statistically relevant in our review [8, 11]

The costs are a very impacting factor in the healthcare system. The majority of them comes from the length of stay, the cost of the implants, and the post-operative complications [25]. In four studies, we found a correlation between increase in costs and malnourishment. The mechanism remains unclear, probably linked to the increase of length of stay, post-operative infection, and other complication [10]. In all the studies, the increase in the mean total of charges of malnourished patient was statistically relevant [11]. An interesting findings are that costs are higher not only during the hospitalization, but also up to 90 day post-operative [1]. Talking about the mean length of stay for a single total joint arthroplasty (TJA), it has seen a dramatic decrease from an average of up to 23 days to the current average of 3.7 days, with several reasons involved [26]. In our review, we found 8 studies that correlated malnourishment with prolonged LOS, mean post-operative length of stay was longer for patient with hypoalbuminemia, in most of them being highly statistically relevant [2, 12]. It is mandatory that every aspect that led to a prolonged LOS, such as hypoalbuminemia, has to be addressed, being a relevant factor for the national care system, but also an impacting social factor as a burden for the relatives in terms of costs, distance, and organization.

Morbidity is a relevant bad outcome after TJA, even if there is an ongoing worldwide temporal decline in mortality following arthroplasty [27]. It is relevant to say that the possible causes of morbidity still have to be addressed. In our review, we found three studies that showed a correlation between morbidity and malnourishment, showing that mortality was significantly higher among the patient with hypoalbuminemia [13], making a hypoalbuminemia a robust and independent risk factor [14].

For all those aspects, the importance of nutritional assessment in elective patient undergoing TJA can no longer be ignored and has to be addressed, his optimization should become a cornerstone in the perioperative management. Only several studies in the last years tried to manage preoperatively malnourished patient with poor results or with insufficient data [20, 28–30], but oral nutrition support strategies (ONSs) (e.g. dietary modification, dietetic counselling, oral nutritional supplements) have been shown to be clinically effective in the management of disease-related malnutrition, but in order to maximize both clinical and cost-effectiveness, it is important to achieve good compliance [31].

Our study has several weak points, first the influence of individual hospital and surgeon quality, case-mix complexity is unknown; secondly, the cut off for low serum albumin (3.5 g/Dl) was set in 1995, and it could be re-examined [10]. In addition, a database bias is present as we used only PubMed and Google Scholar as search engines and also a language bias because we included only paper in English; finally, some of the parameters analysed in each study did not have a clear explanation.

## Conclusion

Although the literature trend indicates that the nutritional status of TJA candidate patients is a parameter that influences the surgical outcome, to the authors’ knowledge, no studies aimed at identifying validated and recognized protocols for the correction of malnutrition.

The improvement and evolution of surgical techniques should go hand in hand with the improvement of overall patient care and its optimization to ensure better clinical results, but also a lower impact on the NHS, meant both as costs and as occupancy of beds.

The low-risk profile and potential benefits of supplements suggest that pre-operative use could play an important role in improving outcomes, but large-scale, polycentric, randomized trials are needed to outline EBM guidelines.

## References

[1] Bala A, Ivanov DV, Huddleston JI, Goodman SB, Maloney WJ, Amanatullah DF (2020). The cost of malnutrition in total joint arthroplasty. J Arthroplasty.

[2] Ihle C, Freude T, Bahrs C, Zehendner E, Braunsberger J, Biesalski HK (2017). Malnutrition—an underestimated factor in the inpatient treatment of traumatology and orthopedic patients: a prospective evaluation of 1055 patients. Injury.

[3] Schroer WC, LeMarr AR, Mills K, Childress AL, Morton DJ, Reedy ME (2019). Chitranjan S. Ranawat award: elective joint arthroplasty outcomes improve in malnourished patients with nutritional intervention: a prospective population analysis demonstrates a modifiable risk factor. Bone Jt J.

[4] Ljungqvist O, Dardai E, Allison SP (2010). Basics in clinical nutrition: perioperative nutrition. e-SPEN.

[5] Inacio MCS, Paxton EW, Graves SE, Namba RS, Nemes S (2017). Projected increase in total knee arthroplasty in the United States—an alternative projection model. Osteoarthr Cartil.

[6] Roche M, Law TY, Kurowicki J, Sodhi N, Rosas S, Elson L (2018). Albumin, prealbumin, and transferrin may be predictive of wound complications following total knee arthroplasty. J Knee Surg.

[7] Moher D, Liberati A, Tetzlaff J, Altman DG, Altman D, Antes G (2009). Preferred reporting items for systematic reviews and meta-analyses: the PRISMA statement. PLoS Med.

[8] Kamath AF, Nelson CL, Elkassabany N, Guo Z, Liu J (2017). Low albumin is a risk factor for complications after revision total knee arthroplasty. J Knee Surg.

[9] Ihle C, Weiß C, Blumenstock G, Stöckle U, Ochs BG, Bahrs C (2018). Interview based malnutrition assessment can predict adverse events within 6 months after primary and revision arthroplasty—a prospective observational study of 351 patients. BMC Musculoskelet Disord.

[10] Rudasill SE, Ng A, Kamath AF (2018). Preoperative serum albumin levels predict treatment cost in total hip and knee arthroplasty. Clin Orthop Surg.

[11] Wilson JM, Schwartz AM, Farley KX, Bradbury TL, Guild GN (2020). Combined malnutrition and frailty significantly increases complications and mortality in patients undergoing elective total hip arthroplasty. J Arthroplasty.

[12] Bohl DD, Shen MR, Kayupov E, Della Valle CJ (2016). Hypoalbuminemia independently predicts surgical site infection, pneumonia, length of stay, and readmission after total joint arthroplasty. J Arthroplasty.

[13] Walls JD, Abraham D, Nelson CL, Kamath AF, Elkassabany NM, Liu J (2015). Hypoalbuminemia more than morbid obesity is an independent predictor of complications after total hip arthroplasty. J Arthroplasty.

[14] Fu MC, Mclawhorn AS, Padgett DE, Cross MB (2013). Hypoalbuminemia is a better predictor than obesity of complications after total knee arthroplasty: a propensity score-adjusted observational analysis. HSS J.

[15] Black CS, Goltz DE, Ryan SP, Fletcher AN, Wellman SS, Bolognesi MP (2019). The role of malnutrition in ninety-day outcomes after total joint arthroplasty. J Arthroplasty.

[16] Kishawi D, Schwarzman G, Mejia A, Hussain AK, Gonzalez MH (2020). Low preoperative albumin levels predict adverse outcomes after total joint arthroplasty. J Bone Jt Surg Am.

[17] Eminovic S, Vincze G, Doris E, Regina R, Patrick S, Andreas L (2021). Malnutrition as predictor of poor outcome after total hip arthroplasty. Int Orthop.

[18] Kamath AF, McAuliffe CL, Kosseim LM, Hume E, Pio F (2016). Malnutrition in joint arthroplasty: prospective stud indicates risk of unplanned ICU admission. Arch Bone Jt Surg.

[19] Fryhofer GW, Sloan M, Sheth NP (2019). Hypoalbuminemia remains an independent predictor of complications following total joint arthroplasty. J Orthop..

[20] Golladay GJ, Satpathy J, Jiranek WA (2016). Patient optimization—strategies that work: malnutrition. J Arthroplasty.

[21] Rai J, Gill SS, Satish Kumar BRJ (2002). The influence of preoperative nutritional status in wound healing after replacement arthroplasty. Orthopedics.

[22] Loftus TJ, Brown MP, Slish JH, Rosenthal MD (2019). serum levels of prealbumin and albumin for preoperative risk stratification. Nutr Clin Pract.

[23] Ban KA, Minei JP, Laronga C, Harbrecht BG, Jensen EH, Fry DE (2017). American College of Surgeons and Surgical Infection Society: surgical site infection guidelines, 2016 update. J Am Coll Surg.

[24] AbdelSalam H, Restrepo C, Tarity TD, Sangster W, Parvizi J (2012). Predictors of intensive care unit admission after total joint arthroplasty. J Arthroplasty.

[25] Maradit Kremers H, Visscher SL, Moriarty JP, Reinalda MS, Kremers WK, Naessens JM (2013). Determinants of direct medical costs in primary and revision total knee arthroplasty knee. Clin Orthop Relat Res.

[26] El Bitar YF, Illingworth KD, Scaife SL, Horberg JV, Saleh KJ (2015). Hospital length of stay following primary total knee arthroplasty: data from the nationwide inpatient sample database. J Arthroplasty.

[27] Zeng C, Lane NE, Englund M, Xie D, Chen H, Zhang Y (2019). In-hospital mortality after hip arthroplasty in China: analysis of a large national database. Bone Jt J.

[28] Gu A, Malahias M, Strigelli V, Nocon AA, Thomas P, Sculco PK (2019). Preoperative malnutrition negatuvely correlates with postoperative wound complication and infection after total joint arthroplasty: a systematic review and meta-analysis. J Arthroplasty.

[29] Burgess LC, Phillips SM, Wainwright TW (2018). What is the role of nutritional supplements in support of total hip replacement and total knee replacement surgeries? A systematic review. Nutrients.

[30] Cao G, Huang Q, Xu B, Huang Z, Xie J, Pei F (2017). Multimodal nutritional management in primary total knee arthroplasty: a randomized controlled trial. J Arthroplasty.

[31] Hubbard GP, Elia M, Holdoway A, Stratton RJ (2012). A systematic review of compliance to oral nutritional supplements. Clin Nutr.

